# Transfer of Silver Nanoparticles through the Placenta and Breast Milk during in vivo Experiments on Rats

**Published:** 2013

**Authors:** E. A. Melnik, Yu. P. Buzulukov, V. F. Demin, V. A. Demin, I. V. Gmoshinski, N. V. Tyshko, V. A. Tutelyan

**Affiliations:** Federal State Budgetary Institution «Institute of Nutrition» of the Russian Academy of Medical Sciences, 2/14 Ust’inski proezd Moscow, 109240, Russian Federation; National Research Center Kurchatov Institute, 123182, Kurchatov sq. 1, Moscow, Russian Federation

**Keywords:** pregnancy, lactation, nanoparticles, radioactive tracer, silver, fetoplacental barrier

## Abstract

Silver nanoparticles (NPs), widely used in the manufacture of various types of consumer products and for medical applications, belong to novel types of materials that pose potential risks to human health. The potential negative effects of the influence of these NPs on reproduction are insufficiently researched. A quantitative assessment of the transfer of metallic silver nanoparticles through the placenta and breast milk was carried out during an *in vivo *experiment. We used 34.9 ± 14.8 nm in size silver NPs that were stabilized by low-molecularweight polyvinylpyrrolidone and labeled with the ^110m^Ag radioactive isotope using thermal neutron irradiation in a nuclear reactor. [^110m^Ag]-labeled NPs preparations were administered intragastrically via a gavage needle to pregnant (20^th^ day of gestation) or lactating (14–16th day of lactation) female rats at a dose of 1.69–2.21 mg/kg of body weight upon conversion into silver. The accumulation of NPs in rat fetuses and infant rats consuming their mother’s breast milk was evaluated using a low-background semiconductor gamma-ray spectrometer 24 and 48 hours following labeling, respectively. In all cases, we observed a penetration of the [^110m^Ag]-labeled NPs through the placenta and ther entry into the mother’s milk in amounts exceeding by 100-1,000 times the sensitivity of the utilized analytical method. The average level of accumulation of NPs in fetuses was 0.085–0.147% of the administered dose, which was comparable to the accumulation of the label in the liver, blood, and muscle carcass of adult animals and exceeded the penetration of NPs across the hematoencephalic barrier into the brain of females by a factor of 10-100. In lactating females, the total accumulation of [^110m^Ag]-labeled NPs into the milk exceeded 1.94 ± 0.29% of the administered dose over a 48 h period of lactation; not less than 25% of this amount was absorbed into the gastrointestinal tract of infant rats. Thus, this was the first time experimental evidence of the transfer of NPs from mother to offspring through the placenta and breast milk was obtained.

## INTRODUCTION

Advances in the development of nanotechnology and the increase in the volume of production and practical application of artificial nanoparticles (NPs) and nanomaterials (NMs) have led to the belief that in the near future NPs could become a significant source of environmental contamination. Among the most prominent NMs, special attention is focused on the silver NPs that are widely used in various types of consumer products (disinfecting agents, textiles, paint-and-lacquer materials, cosmetics, packaging materials, food supplements) [1, 2] and for a variety of biopharmaceutical applications, including their use as antimicrobial [3, 4], anti-inflammatory agents [5] and as *in vivo *molecular nanodiagnostic tools [6]. However, silver NPs should also be regarded as a particular source of risks because of their potential toxicity to humans [7-15]. This particularly applies to those risks that are associated with the influence of NMs on a child’s organism as a result of their possible transfer across the placenta and through breast milk [16]. The possibility of a transfer of the NMs present in a mother’s diet or used by her as part of cosmetic products or household chemical products to her offspring cannot be excluded [17]. A quantitative evaluation of such a transfer of NPs is necessary in order to gauge the potential risks of exposure of offspring to the silver NPs in their mother’s body and develop appropriate protective measures, including hygiene regulation of consumer products.

However, the very idea of a natural transfer of the NPs entering a mother’s body to her offspring has yet to be sufficiently investigated. This is due to the specific methodological difficulties associated with detecting the presence of NPs in biological objects [18, 19]. An analysis of the methods used to detect NPs in biological samples, including electronic and atomic force microscopy, spectroscopic methods, chromatography, the use of fluorescent, spin, stable-isotope and other labels [18], has enabled to pinpoint the radioactive tracer technique as the optimal method. The latter is strictly quantitative, highly sensitive, and enables a simple and graphic interpretation of the results related to NPs which do not undergo biotransformation and biodegradation in the body – gold, platinum, and silver NPs [20].

The present work contains a quantitative assessment of the transport of silver NPs through the placenta and breast milk on a model of pregnant and lactating female rats using [^110m^Ag]-labeled NPs preparations.

## EXPERIMENTAL

**Experiment design**

The study was conducted on pregnant and lactating Wistar rats at the clinic of laboratory animals of the Federal State Budgetary Institution “Institute of Nutrition” of the Russian Academy of Medical Sciences. During the preconception period and throughout the pregnancy and lactation, the females received the standard semi-synthetic diet according to [21]. The gestation period of the females was 20 days following conception; lactation period – on average 10–11 days after the birth of offspring. Pregnant rats received [^110m^Ag]- labeled silver NPs intragastrically through a gavage needle at a dose of 1.69 mg/kg of body weight (three females) and 2.21 mg/kg of body weight (four females) in the form of a dispersion in deionized water containing a non-toxic, non-absorbable in the gastro-intestinal tract (GIT) stabilizer of NPs – polyvinylpyrrolidone (PVP) with a molecular weight of 15-30 kDa. The rats were then placed into individual cages made of polystyrene. Twenty-four hours following the administration of the preparation, the rats were subjected to deep anesthesia using diethyl ether, their abdominal cavities were dissected, the rats were bled from the inferior vena cava, and the uterus with fetuses, the liver, and brain were collected. The fetuses were removed from the uterus and thoroughly washed to get rid of the amniotic fluid. Thereafter, the fetuses, liver, and brain of the females were placed into vials made of high-purity polyethylene for gamma spectrometry. Precaution measures to avoid contamination of the organs and fetuses of the rats with NPs in other internal organs and blood were observed during sampling.

In the experiment on lactating rats, five female species nursing 9 infant rats each were administered a solution of [^110m^Ag]-labeled silver NPs intragastrically at a dose of 2.11 mg/kg of body weight. The rats were then returned to their individual cages made of polystyrene, where their offspring were located. According to the conditions of the experiment, the possibility of consumption of female excrements (coprophagy) by infant rats was excluded. Forty eight hours after labeling, infant rats nursed by the females were subjected to a lethal dose of diethyl ether by inhalation. They were then thoroughly washed to remove any traces of female excrements from the fur; the skin with subcutaneous fat tissue was removed, and carcasses were placed into vials for gamma spectrometry. The carcasses of the four infant rats were dissected; the gastrointestinal tract, liver, kidneys, spleen, and the remaining carcass were removed. The preparations obtained were placed separately into vials for gamma spectrometry.

**Obtaining [^110m^Ag]- labeled nanoparticles**

**Fig. 1:**
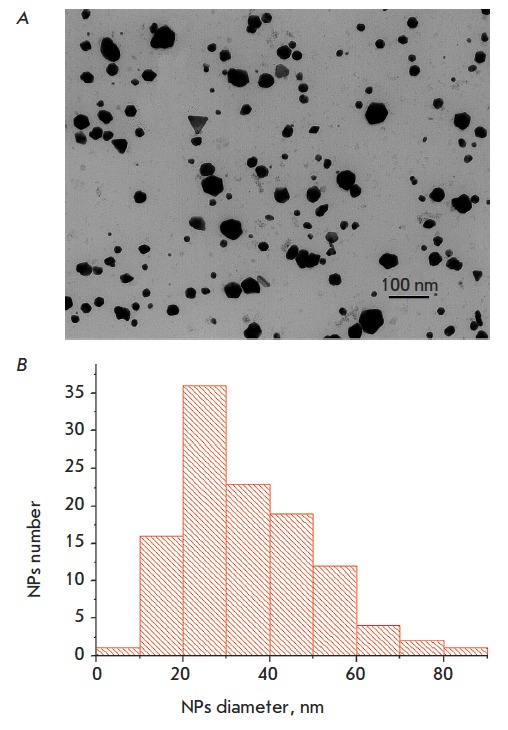
Transmission electronic microscopy of silver NPs preparation Argovit®. NPs image (A) and diameter distribution (B). Electron microscope JEM-100CX (“Jeol”, Japan), accelerating voltage 80 kV. Data obtained by prov., D.Sc. Dzantiev B.B., RAS Institute of biochemistry, Moscow

A preparation of colloidal silver “Argovit” produced by the Scientific and Production Company “Vector-Vita,” Co., Ltd. (Russia) was used for the experiments. The preparation is an aqueous dispersion of NPs of metallic silver containing 1.0–1.4% of silver and 18.6–19.0% of PVP by weight. According to electron microscopy data (*Fig. 1*), the average diameter of the NPs was 34.9 ± 14.8 nm; the minimum size was 8.4 nm, and maximum size was 80.9 nm; the particle’s shape was close to spherical. The preparation was diluted with deionized water at a ratio of 1:11 or 1:47 and sealed in high-purity quartz vials which were then subjected to thermal neutron irradiation (0.005 < En < 0.4 eV) in the vertical experimental canal VEC -9 of the nuclear reactor IR-8. After removal from the reactor, the vials were kept for 48 hours to reduce the background gamma activity of the short-lived silicon isotope in the vial material, followed by their opening. The contents were pooled, sonicated (5 min, 44 kHz, 40 W) to eliminate secondary aggregation of NPs, and adjusted to a fixed volume using deionized water. A total of 0.04 ml of the dispersion of [^110m^Ag]-labeled NPs was collected, transferred into vials for gamma spectrometry, and adjusted using deionized water to a volume approximately corresponding to the volume of the biological sample (fetus or carcass of the infant rat) immediately prior to the administration radioisotopic label (μ) in % of the injected dose according to the equation:

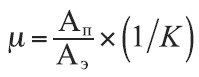



where A_p_ is the bioprobe activity, A_r_ is the activity of the reference sample containing 0.04 cm^3^ of the dispersion of the [^110m^Ag]-labeled NPs, and *K *is the conversion factor representing the ratio between the individual amount of the dispersion of NPs (cm^3^/kg body weight) administered to the females and the average dose obtained by dividing the total volume of the administered dispersion by the total body weight of all female rats in the group.

The concentration of silver NPs in the analyzed samples expressed in ng/g of the sample was calculated with allowance for the individual amount of NMs administered to the female according to the following equation:

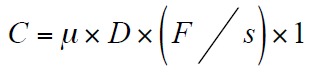



where μ is the relative amount of the radioactive label, % of the amount administered to the female rat, D is the administered dose in mg/kg of body weight, F is the female rat body mass in kg, s is the mass of the biological sample in g, and 106 is the coefficient of transition from mg to ng.

Application of the relative gamma-spectrometric measurements technique for determining the mass of ^110m^Ag in the biological samples enabled to exclude the absolute activity (expressed in Bq) from the calculations and use primary raw data of measurements (impulses/ sec), thus, eliminating a number of errors that had occured during the transition from the primary raw data to absolute activity. In this situation, the basic error of measurements is determined by the background values in the selected energy range. For this reason, the concept of minimum detectable activity (MDA) in the form of a limit of quantitative determination, *L*_q_, was utilized for assessing the minimum significant level of the ^110m^Ag counting rate in the samples [23, 24].

**Metrological characteristics of the method**

For determining the metrological characteristics we utilized the concept of MDA in the form of the limit of quantitative determination, *L*_q_, calculated according to the relation:

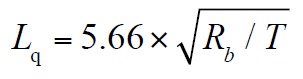



where *R_b_*is the background counting rate equal to 2.64 X 10^-3^ impulses/sec in the applied gamma-spectrometric devices, *T *is the average measurement time of the sample equal to 3600 sec, and 5.66 is the coefficient with allowance for the confidence interval of evaluation of *p *= 0.95 and relative statistical uncertainty ± 50%. Accordingly, *L*q was equal to 4.8 X 10^-3^ impulses/sec [23, 24]. With regard to the preparation used, [^110m^Ag]-NPs corresponds to the minimum limit of quantitative determination of 2.6 ng of silver NPs.

## EXPERIMENTAL

**Fig. 2:**
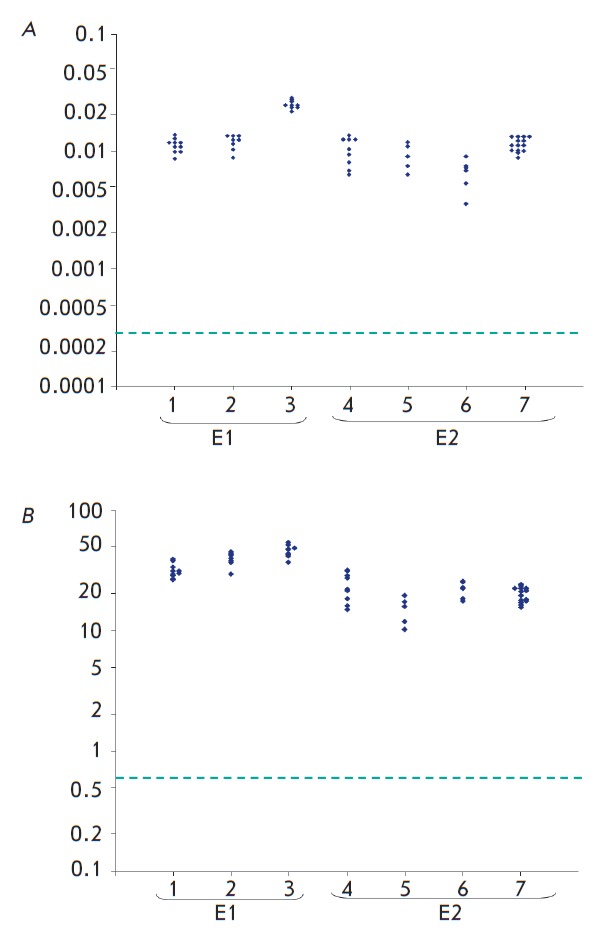
Individual values of total content (A) and concentration (B) of [^110m^Ag]-NPs in fetuses of rats. Axis of abscises – № of female, № of experiment. Ordinate axis – NPs content, % of dose ingested (A) or concentration, ng/g tissue (B). Dotted line marks the threshold of quantitative determination of [^110m^Ag]-NPs in samples

Figure 2 shows the content and the concentration of Ag NPs in rat fetuses 24 hours after intragastric administration of [^110m^Ag]-labeled NPs to pregnant females, ;and *Table 1*shows the mean (*M *± *m*) values per each pregnant female rat and for the experiment in general with respect to two utilized doses of NMs. As can be seen from these results, silver NPs were identified in the fetuses of all pregnant females in amounts significantly exceeding the detection limit. The findings are indicative of penetration of silver NPs through the intestinal wall and placenta with subsequent accumulation in the fetuses.

**Table 1:** Results of silver [^110m^Ag]-NPs determination in fetuses from pregnant rats after 24 hours of intragastric administration of labeled preparation

№ experiment	Dose mg per kg body mass of female	№№ females	Number of fetuses	Mean, M±m		
				Total NPs content in single fetus, % of ingested dose	NPs concentration in fetus, ng/g of tissue	Mass of fetus, g
1	1.69	1	10	0.0114±0.0005	31.7±1.4	2.66±0.04
		2	8	0.0122±0.0006	40.0±1.8	2.13±0.08
		3	9	0.0254±0.0007	46.7±1.8	4.07±0.08
		Mean of 1-st experiment (N=27)		0.0163±0,013	39.1±1.5	2.97±0.16
		Test of homogenity for rats №№ 1-3 ANOVA, P		< 0.001	< 0.001	< 0.001
2	2.21	4	9	0.0104±0.0009	23.7±2.2	3.91±0.08
		5	5	0.0093±0.0011	15.1±1.8	5.72±0.10
		6	6	0.0067±0.0008	22.2±1.4	3.07±0.23
		7	14	0.0116±0.0004	20.1±0.8	5.26±0.10
		Mean of 2-nd experiment (N=34)		0.0101±0.0005	20.7±.08	4.58±0.18
		Test of homogeneity for rats №№ 4-7 ANOVA, P		< 0.001	0.008	< 0.001

Comparison with data on the absorption and interorgan distribution of [^110m^Ag]-labeled NPs administered intragastrically to male rats at a comparable dose (0.81 mg of Ag/kg of body weight) [20] shows (*Table 2*) that penetration of Ag NPs through the placenta exceeds the accumulation in the brain by more than 10 times, corresponds to the level in the blood and spleen, and is significantly lower than the accumulation in the liver. The silver NPs content in the liver and brain of pregnant female rats determined in the present experiment (*Table 2*) did not differ significantly from the values obtained previously for adult males [20] under comparable conditions (P > 0.05; *t*-Student test).

**Table 2:** Comparison of mean NPs accumulation in fetuses and in internals of pregnant rats 24 hours after intragastric administration of [^110m^Ag]-NPs

Experiment №	Dose of ([^110m^Ag]-NPs), mg/kg body mass	Number of rats	Organ/tissue	Content, % of dose ingested
Male rats, experiment from 2011 [11]	0.81	4	Carcass	0.36±0.17
			Liver	0.60±0.18
			Blood	0.126±0.051
			Spleen	0.054±0.020
			Testes	0.016±0.003
			Kidneys	0.014±0.002
			Lungs	0.0094±0.0026
			Brain	0.0029±0.0010
			Pancrestic	0.0079±0.0015
			Heart	0.0042±0.0016
Pregnant females, present study	1.69	3	Fetuses in total	0.147±0.041
			Liver	0.559±0.229
			Brain	0.0035±0.0004
	2.21	4	Fetuses in total	0.085±0.028
		4	Liver	0.324±0.046
		4	Brain	0.0035±0.0006

**Fig. 3:**
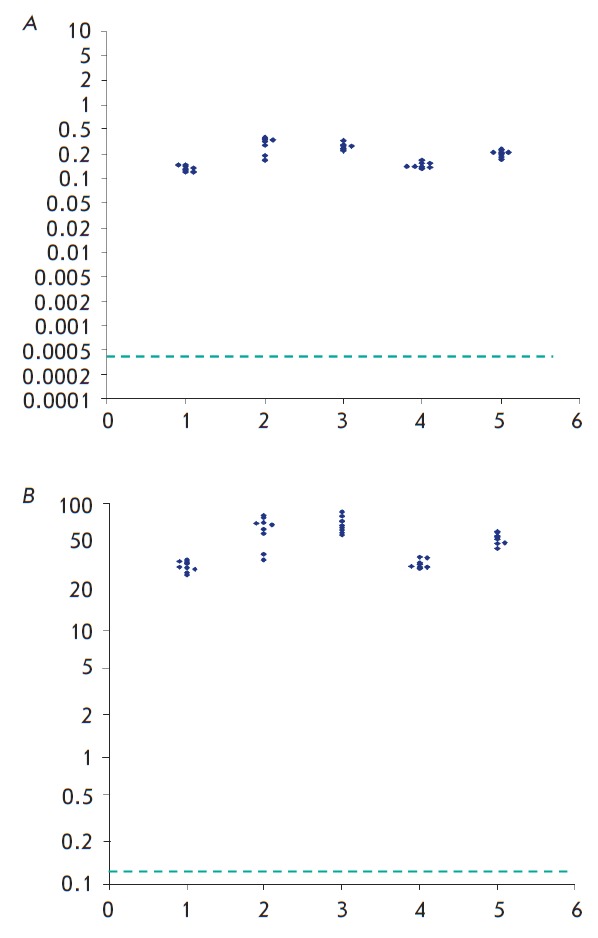
Individual values of total content (A) and concentration (B) of [^110m^Ag]-NPs in suckling pups of rats. Axis of abscises –№№ of nursing dams, № of experiment. Ordinate axis – NPs content, % of dose ingested (A) or concentration, ng/g tissue (B). Dotted line marks the threshold of quantitative determination of [^110m^Ag]-NPs in samples

As can be seen from the data presented in *Fig. 3* and *Table 3, *[^110m^Ag]-labeled NPs administered intraperitoneally to lactating females were detected in the bodies of all 45 infant rats in the litters of five lactating rats. The concentrations of these NPs significantly (100-100-fold) exceeded the limit of quantitative determination.

**Table 3:** Results of silver [^110m^Ag]-NPs determination in suckling pups 48 hours after intragastric administration to nursing dams.

№ experi- ment	Dose mg per kg body mass of female	№№ females	Number of pups	Mean, M±m		
				Amount of [^110m^Ag]-NPs in one suckling pup, % of ingested dose	Concentration [^110m^Ag]-NPs in pups ng/g body weight	Mass of pups, g
1	2.11	1	9	0.136±0.004	31.9±1.0	28.9±0.4
		2	9	0.302±0.022	62.5±5.2	29.2±0.4
		3	9	0.272±0.009	68.5±3.2	28.2±0.8
		4	9	0.150±0.004	33.3±0.9	31.4±0.7
		5	9	0.220±0.007	52.9±1.7	28.5±0.2
		Mean of experiment (N=45)		0.216±0.011	49.8±2.6	29.3±0.3
		Test of homogenity for rats №№ 1-5, ANOVA, P		< 0.001	< 0.001	0.002
		Total content in offspring, % of ingested dose		1.94±0.29		

According to the experimental conditions, such amounts of [^110m^Ag]-labeled NPs cannot be explained by ingestion of female feces containing significant amounts of NPs by the offspring, contamination of the cutaneous covering removed prior to carrying out the gamma-ray spectrometry, and ingestion of the mat contaminated with female rat urine, as the total excretion of silver NPs with urine as follows from the data [20] did not exceed 0.032% of the administered dose of the preparation over 2 days, which was 60 times less than the total amount of NPs detected in infant rats.

As can be seen from *Table 4*, the maximum amount of ^110m^Ag was detected in the gastro-intestinal tract of infant rats; however, significant (well above the limit of quantitative determination) levels of nanoparticles were detected in the internal organs and in the carcasses of the infant rats, which in turn indicates a high level of absorption of NPs in their GIT.

**Table 4:** Tissue distribution of [^110m^Ag]-NPs in suckling pups (N=4) 48 hours after intragastric administration to nursing dams

№№	Organ/ tissue	Mean content of [^110m^Ag]-NPs , % of amount, detected in the pup, M±m	Mean content of [^110m^Ag]-NPs , % dose ingested by nursing dams, M±m
1	GIT	73.8±4.4	0.106±0.006
2	Carcass	7.4±1.4	0.0125±0.0011
3	Liver	17.9±3.0	0.0287±0.0033
4	Kidney	0.90±0.18	0.0014±0.0003

As follows from *Table 3*, the total amount of [^110m^Ag]- labeled NPs excreted with milk and detected in infant rats was comparable (or exceeded) to the one-time total content of the label in all organs and the carcass of the animal following intragastric administration of the preparation (provided in *Table 2*according to [20]). Therefore, there are grounds to believe that the excretion of Ag NPs with milk during lactation is one of the major ways of excreting these nanoparticles from the body, which is second only to fecal excretion and is far superior to urinary excretion with respect to quantity.

## DISCUSSION

Thus, the concentration of NPs in organs and tissues of the offspring is equal to approximately 0.020–0.040 and 0.030–0.070 μg/g of tissue, respectively, upon administration of Ag NPs at doses of approximately 2 mg/kg of body weight to pregnant and lactating female rats. The doses of silver NPs administered to female rats were relatively high upon conversion to average human body weight (70 kg) and were approximately 140 mg. The possibility of exposure of a human to such quantities of NMs at the same time may occur upon consumption of contaminated drinking water, food products, or abuse of Ag-containing FS. The data obtained confirm directly the feasibility of transfer of silver NPs entering the gastrointestinal tract of the mother to her offspring during pregnancy and lactation. The likelihood of such transport of NPs of various types has repeatedly been postulated as a potential source of risks to the development of a fetus and newborn [16, 17], although direct experimental evidence for the occurrence of the process is scarce. It was demonstrated [25] that 14 nm in size silver NPs are absorbed in the gastrointestinal tract of adult rats in limited amounts in the course of multiple intragastric administrations over a period of 28 days and are distributed between organs and tissues, including kidneys and the liver. Data on the penetration of silver NPs through the fetoplacental barrier and mammary gland are unavailable; however, results confirming the transfer of similar, with respect to physical and chemical properties, 12-14 nm in size metal nanoparticles made of gold [26] following their intravenous administration to pregnant female mice have been obtained. Penetration of CdSe quantum dots through the fetoplacental barrier after parenteral administration to female mice was described in [27]; the ability of 50-100 nm in diameter fluorescent polystyrene NPs to penetrate the fetoplacental barrier modeled by a monolayer of human choriocarcinoma cells was demonstrated in [28]. Our data demonstrate that with respect to silver NPs the process is carried out *in vivo *under conditions of natural route of entry of NPs into a mother’s body.

The question arises as to how significant are the concentrations of NPs detected in rat fetuses and infant rats and whether they can pose a hazard to the development and health of the offspring. A relatively substantial amount of data has been accumulated on the biological effects of silver NPs given different ways of* in vivo *administration. Thus, colloidal silver was administered to mice intraperitoneally at extremely high doses approaching 1000 mg/kg [12]. Under these deliberately non-physiological conditions, NPs managed to penetrate the hematoencephalic barrier, causing the development of signs of oxidative stress in various regions of the brain. The genotoxic effects of silver NPs administered intraperitoneally at a dose of approximately 1 mg/kg of body weight to mice were demonstrated in [13]. Interpretation of the results of this work was rendered difficult by the presence of toxic surfactants in the NPs preparation – dioctyl sodium sulfosuccinate. The presence of inhalation toxicity by silver NPs in rats was established [14, 15]. Oral administration of this nanomaterial at a dose approaching 30 mg/kg of body weight to rats over 28 days resulted in no signs of systemic toxicity or genotoxic effects in the rats, although silver NPs accumulated in the kidneys and liver of the animals [29]. Significantly higher doses of silver nanoparticles (approaching 1000 mg/kg of body weight) administered orally resulted in the emergence of specific biochemical and histopathological changes indicative of toxicity [8, 29].

The toxic properties of silver NPs rendered important the assessment of the likelihood of toxicity in the offspring of animals subjected to exposure to this substance as a result of transplacental transfer or transfer through milk. The data on the *in vitro *cytotoxicity of silver NPs obtained under conditions when the concentration of NPs is precisely determined is without doubt of interest. Thus, it was demonstrated that silver NPs at a concentration of 5–50 μg/cm^3^ damage cultured BRL3A rat hepatocytes [30]. The cytotoxic effects of silver NPs identifiable by the release of lactate dehydrogenase in a mitochondrial tetrazolium test were demonstrated at concentrations exceeding 5 μg/cm^3^ in experiments on rat spermatogonial cells [31]. Stimulation of apoptosis in mouse fibroblasts was also observed (using caspase-3 activity assay) at a concentration of silver NPs exceeding 3.12 μg/cm3 [32]. Silver NPs at a concentration exceeding 10 μg/cm^3^ impaired the conductivity for Na^+^ ions in cultured CA^-1^ hippocampal neurons [33]. Experiments on mononuclear cells of human peripheral blood [34] demonstrated that silver NPs at a concentration equal to or exceeding 3 μg/cm3 stimulate the production of the tumor necrosis factor-α. A pronounced cytotoxic effect of silver NPs was observed at concentrations exceeding 15 μg/cm^3^. According to [35], silver NPs coated with PVP or citrate are capable of influencing the differentiation of PC12 pheochromocytoma cells of neuroendocrine origin. The minimum effective concentration of NPs was 3 μM by silver (approximately 0.3 μg/cm^3^). Finally, the effects of different in size silver NPs in the primary culture of rat cortical neurons were characterized in [36]. A statistically significant increase in the death of cells cultured for 14 days in the presence of 20 nm in size NPs at a minimum concentration equal to or exceeding 5 μg/cm^3^ was demonstrated. The toxicity of NPs decreased with a decrease in size. Thus, 40 nm in diameter NPs were only cytotoxic at a concentration exceeding 10 μg/cm^3^.

A comparison of the data provided above with the results of our work enables to suggest that the concentration of silver NPs in rat fetuses (not exceeding 50 ng/g of tissue at an administered dose of NPs of approximately 2 mg/kg of body weight, *Table 1*) were 60–300 times lower than the minimum effective concentrations of NPs detected in *in vitro *systems. However, this estimate does not take into account the possibility of a non-uniform distribution of NPs between the organs and tissues of the fetus. It is known that silver NPs accumulate mainly in the liver and kidneys [20, 25]. If we assume that all nanomaterials accumulate in one of these organs, whose mass at this gestational age is 6.0 and 0.9% of the fetal weight, then we obtain an excessive concentration of NPs in the organs – 830 and 5000 ng/g in the liver and kidneys, respectively. The latter value is comparable to an *in vitro *determined lower limit of a possible cytotoxic effect equal approximately to 3000–5000 ng/g. It should be noted that the dose of nanomaterials administered to the pregnant female rats of approximately 2 mg/kg of body weight was aggravated by a factor of 2,000 in comparison with the upper tolerable level of silver intake in any form (colloidal particles and ions), which is equal to 70 μg or approximately 1 μg/kg of human body weight. It can, therefore, be concluded that the level of accumulation of silver NPs in the organs of rat fetuses subject to certain conditions can be regarded as safe in the event of intake of silver nanoparticles in physiological amounts (e.g., together with drinking water or food supplements).

The average level of labeled NPs in infant rats receiving milk feeding was 50 ng/g. Seventy-five percent of this value is attributed to the label detected in the gastrointestinal tract. The content of NPs in the liver amounts to 17.9%, and in the kidneys it amounts to 0.9% of the total amount detected in an infant rat. The mass of organs at this age of development is equal to 3.8 and 1.2% of body weight on average, which implies that the concentration of NPs in these organs is approximately 235 and 38 ng/g, respectively. These values are well below the hypothesized level at which cytotoxic effects can be observed and are indicative of the safety of the intake of NPs by lactating females at the above mentioned, deliberately aggravated dose to the development of the offspring.

Therefore, on the basis of the published data it may be concluded that the levels of NPs in the tissues of infant rats and fetuses detected after a single-dose administration of NPs to female rats can be regarded as safe. At the same time, the following must be given due consideration: firstly, the possible accumulation of NPs in the body upon multiple intakes; hence, the level of NPs in the organs and tissues will be higher than upon a single instance of intragastric exposure, and, secondly, partial matching of the conditions during *in vitro *experiments and *in vivo*. In particular, the duration of the exposure to NPs in cell cultures amounts to hours, rarely – 7–14 days, while *in vivo *their effects may last for a lifetime. Therefore, the study of reproductive toxicity must be recommended during a comprehensive assessment of the safety of novel types of NPs and NMs. The transfer of NPs through the placenta and breast milk should be considered during the development of procedures aimed at maximizing the prevention of exposure of a woman’s body to NPs and NMs during pregnancy and lactation.

## CONCLUSIONS

1. It was established that [^110m^Ag]-NPs penetrate the placenta and reach breast milk in quantities exceeding the sensitivity of the analytical method used by a factor ranging from 100 to 1,000 upon administration of ^110m^Ag-labeled silver NPs into the gastrointestinal tract of pregnant and lactating female rats at a dose of approximately 2 mg/kg of body weight.

2. The average level of accumulation of NPs in fetuses was 0.085–0.147% of the administered dose, which was comparable to the accumulation in the liver of female rats (0.3–0.5% of the administered dose) and exceeded the penetration of NPs through the hematoencephalic barrier into the brain of female rats by at least 10-100 times (3.5.X 10^-3^ %).

3. In lactating females the total inflow of [^110m^Ag]-NPs into the milk was no less than 1.94 ± 0.29% of the administered dose over a 48-hour period of lactation; no less than 25% of the amount was absorbed in the digestive tract of infant rats.

Maximum levels of silver NPs were detected in the kidneys of fetuses upon their administration to female rats at a dose multiplied 2,000 times in comparison with an adequate level of intake of this microelement, where they were not significantly higher than the toxic concentrations established during *in vitro *experiments; in other cases, the levels of NPs were significantly lower than the effective concentrations. However, considering the possible effects of an accumulation of NPs in the organs and tissues of offspring upon their prolonged intake by the mother, it is recommended to conduct an investigation into the reproductive toxicity of NPs in the course of a comprehensive assessment of their safety.

Therefore, for the first time experimental evidence of the transfer of silver NPs from a mother to her offspring through the placenta and breast milk has been obtained.

## Abbreviations

FS: food supplement
GIT: gastrointestinal tract
MG: methodological guidelines
MDA: minimum detectable activity
MPs: nanoparticles
NMs: nanomaterials
PVP: polyvinylpyrrolidone
M: sample mean
m: standard error of the mean

## References

[1] Vernikov V.M., Gmoshinski I.V., Khotimchenko S.A. Voprosy pitanija. (2009). Problems of nutrition.

[2] Blaser S.A., Scheringer M., Macleod M., Hungerbühler K. (2008). Sci. Total Environ..

[3] Fayaz M.A., Ao Z., Girilal M., Chen L., Xiao X., Kalaichelvan P.T., Yao X. (2012). Int. J. Nanomedicine..

[4] Acosta-Torres L.S., Mendieta I., Nuñez-Anita R.E., Cajero-Juárez M., Castaño V.M. (2012). Int. J. Nanomedicine..

[5] Bhol K.C., Schechter P.J. (2007). Dig. Dis. Sci..

[6] Chrastina A., Schnitzer J.E. (2010). Int. J. Nanomedicine..

[7] Shumakova A.A., Smirnova V.V., Tananova O.N., Trushina E.N., Kravchenko L.V., Aksenov I.V., Selifanov A.V., Soto G.S., Kuznetsova G.G., Bulachov A.V., Safenkova I.V., Gmoshinski I.V., Khotimchenko S.A. (2011). Voprosy pitanija (Problems of nutrition)..

[8] Kim Y.S., Song M.Y., Park J.D., Song K.S., Ryu H.R., Chung Y.H., Chang H.K., Lee J.H., Oh K.H., Kelman B.J., Hwang I.K., Yu I.J. (2010). Subchronic oral toxicity of silver nanoparticles. Part Fibre Toxicol..

[9] Wijnhoven S.W.P., Peijnenburg W.J.G.M., Herberts C.A., Hagens W.I., Oomen A.G., Heugens E.H.W., Roszek B., Bisschops J., Gosens I., Van De Meent D., Dekkers S., De Jong W.H., Van Zijverden M., Sips A.J.A.M., Geertsma R.E. (2009). Nanotoxicology..

[10] Onischenko G.G., Archakov A.I., Bessonov V.V., Bokit’ko B.G., Gintsburg A.L., Gmoshinski I.V., Grigor’ev A.I., Izmerov N.F., Kirpichnikov M.P., Naroditsky B.S., Pokrovsky V.I., Potapov A.I., Rakhmanin Yu.A., Tutelyan V.A., Khotimchenko S.A. (2007). Gigiena i Sanitaria (Hygiene and Sanitation)..

[11] Onischenko G.G., Tutelyan V.A. (2007). Voprosy pitanija (Problems of nutrition)..

[12] Rahman M.F., Wang J., Patterson T.A. (2009). Expression of genes related to oxidative stress in the mouse brain after exposure to silver-25 nanoparticles. Toxicol. Lett..

[13] Ordzhonikidze C.G., Ramaiyya L.K., Egorova E.M., Rubanovich A.V. (2009). Genotoxic Effects of Silver Nanoparticles on Mice in Vivo. Acta naturae..

[14] Sung J.H., Ji J.H., Yoon J.U. (2008). Lung function changes
in Sprague-Dawley rats after prolonged inhalation exposure
to silver nanoparticles. Inhal. Toxicol..

[15] Ji J.H., Jung J.H., Kim S.S. (2007). Twenty-eight-day inhalation toxicity study of silver nanoparticles in Sprague-Dawley rats. Inhal. Toxicol..

[16] Oberdörster G., Maynard A., Donaldson K., Castranova V., Fitzpatrick J., Ausman K., Carter J., Karn B., Kreyling W., Lai D., Olin S., Monteiro-Riviere N., Warheit D., Yang H. (2005). Part. and Fibre Toxicol..

[17] Yokel R.A., MacPhail R.C. (2011). J. Occupational Med. Toxicol..

[18] Raspopov R.V., Gmoshinski I.V., Popov K.I., Krasnoyarova O.V., Khotimchenko S.A. (2012). Voprosy pitanija (Problems of nutrition)..

[19] Tiede K., Boxall A.B., Tear S.P., Lewis J., David H., Hassellov M. (2008). Food Add.Contam..

[20] Buzulukov Yu.P., Gmoshinski I.V., Raspopov R.V., Demin V.F., Soloviev V.YU., Kuzmin P.G., Shafeev G.A., Khotimchenko S.A. (2012). Medical Radiol Radiat Safety.

[21] Tyshko N.V., Zhminchenko V.M., Pashorina V.A., Selyaskin K.E., Melnik E.A., Mustafina O.K., Soto S.G., Trushina E.N., Gapparov M.M.G. (2011). Voprosy pitanija (Problems of nutrition)..

[22] IAEA Database on www.iaea.org/OurWork/NuclearDataService..

[23] Isaev A.G., Babenko V.V., Kazimirov A.S., Grishin S.I., Ievlev S.M. (2010). Problems of nuclear power stations and Chernobyl safety.Kiev..

[24] Lochamy J.C. (1981). The Minimum Detectable Activity Concept.. EG&G ORTEC Systems Application Studies, PSD,September 1981.

[25] Loeschner K., Hadrup N., Qvortrup K., Larse A., Gao X., Vogel U., Mortensen A., Lam H.R., Larsen E.H. (2011). Part. Fibre Toxicol..

[26] Yang H., Sun C., Fan Z., Tian X., Yan L., Du L., Liu Y., Chen C., Liang X., Anderson G.J., Keela J.A., Zha Y., Nie G. (2012). Sci.Reports..

[27] Chu M., Wu Q., Yang H., Yuan R., Hou S., Yang Y., Zou Y., Xu S., Xu K., Ji A., Sheng L. (2010). Small..

[28] Cartwright L., Poulsen M.S., Nielsen H.M., Pojana G., Knudsen L.E., Saunders M., Rytting E. (2012). Int. J. Nanomedicine..

[29] Kim Y.S., Kim J.S., Cho H.S., Rha D.S., Kim J.M., Park J.D., Choi B.S., Lim R., Chang H.K., Chung Y.H., Kwon I.H., Jeong J., Han B.S., Yu I.J. (2008). Inhal. Toxicol..

[30] Hussain S.M., Hess K.L., Gearhart J.M., Geiss K.T., Schlager J.J. (2005). Toxicol. in Vitro..

[31] Braydich-Stolle L., Hussain S., Schlager J.J., Hofmann M.C. (2005). Toxicol. Sci..

[32] Arora S., Jain J., Rajwade J.M., Paknikar K.M. (2009). Toxicol. Appl. Pharmacol..

[33] Liu Z., Ren G., Zhang T., Yang Z. (2009). Toxicology..

[34] Shin S.H., Ye M.K., Kim H.S., Kang H.S. (2007). Int.Immunopharmacol..

[35] Powers C.M., Badireddy A.R., Ryde I.T., Seidler F.J., Slotkin T.A. (2011). Environ. Health Perspect..

[36] Haase A., Rott S., Mantion A., Graf P., Plendl J., Thünemann A.F., Meier W.P., Taubert A., Luch A., Reiser G. (2012). Toxicol. Sci..

